# Attitudes of Italian mental health nurses towards mental illness and recovery: a cross-sectional study

**DOI:** 10.3934/publichealth.2023025

**Published:** 2023-05-06

**Authors:** Giovanni Napoli, Simone Autuori, Kumi Senkyire Ephraim

**Affiliations:** 1 Dipartimento di Salute Mentale, Azienda Sanitaria Universitaria Friuli Centrale (ASUFC), Udine, Italy; 2 UOC Psichiatria 2, Azienda Ospedale-Università Padova (AOUP), Padova, Italy; 3 Ga West Municipal Hospital-Ghana Health Service, Accra, Ghana

**Keywords:** mental health, nursing, psychiatric nursing, stigma, recovery

## Abstract

**Background:**

Mental health nurses' (MHNs) stigma and discrimination against people with mental illnesses are obstacles to recovery and the development of effective care and treatment. Although many authors have been interested in exploring stigma among general health professionals, paradoxically, less and non-generalizable evidence is available on this phenomenon among MHNs. Understanding the factors associated with stigma and its relationship to recovery attitudes among MHNs could allow for more accurate interventions and improve patient care outcomes.

**Objective:**

This study conducted on a sample of Italian psychiatric nurses had the objective of analyzing the aptitude for recovery and the tendency towards stigma of these professionals towards mental illness.

**Methodology:**

A cross-sectional web survey was conducted on a sample of Italian MHNs, who were administered two validated tools, the RAQ-7 (assessment of recovery aptitude) and the WHO-HC-15 (assessment of stigma) respectively.

**Results:**

A total of 204 MHNs were interviewed. The analysis showed positive overall scores (high recovery aptitude and low stigma levels) among participating MHNs. The attitude to recovery appeared to be directly related to a lower tendency to stigma towards mental illness. It has been observed that MHNs with advanced levels of education appear to be more predisposed to recovery, as well as generally less stigmatizing. There is evidence that the setting in which care is provided, marital status and age can play a significant role in the tendency to stigmatization.

**Conclusion:**

Our manuscript could assist nursing executives, leaders or educators in making decisions about managing and preventing stigma among MHNs.

## 1. Introduction

The “stigma” is a sociocultural phenomenon that extends into various fields starting from ancient Greece, in fact this noun indicated the tattoos that criminals wore to be identified and consequently avoided, mainly in public places [1]. The Canadian sociologist Erwin Goffman, theorized the concept of stigma, adopted by social psychiatry, to define the set of negative prejudices attributed to people with mental problems due to their disorder and which lead to rejection, discrimination and exclusion [2]. In this regard, it was found that stereotypes towards people with psychiatric illness tend to be associated with dangerousness, responsibility for the disease, inability caused by the same and reduced intelligence [3].

The stigma that society projects on individuals with (external) mental illness is often internalized and assimilated by the latter, generating a maladaptive process called self-stigma [4], through which the person accepts social prejudices and integrates into one's self-concept [5]. The self-stigma can generate in the person with psychiatric illness, the aggravation of the symptoms of the disease, the non-adherence to therapeutic treatments and the renunciation of an autonomous (working / sentimental) life, as the social prejudices are assumed by the person as true, resulting in the loss of confidence in the possibility of healing [6]–[9]. Stigmatization and discrimination therefore represent a great barrier to the healing process of the individual suffering from mental pathology [10] and re-integration into society [11]. Despite various initiatives and campaigns against stigma in mental health [12]–[14], hostile and stigmatizing attitudes still exist towards people with mental illness especially in the general population [15]–[17]. Among health professionals, although some authors have shown that psychiatric nurses generally show more positive attitudes towards patients with mental illness than the general public and general practitioners [18]–[21], the data nevertheless revealed negative attitudinal characteristics within this nursing category [22],[23]. This condition appears to be in contrast with the presuppositions of recovery in psychiatry, which aims to make patients acquire a lifestyle independent of taking charge by mental health services, characterized by housing and working stability, by enriching social relations and a process of individual and organizational empowerment [24],[25]. In this sense, the pursuit of the fight against stigma (both external and self-stigma) represents a cornerstone of recovery-oriented mental health services [26]–[28].

Although there is much evidence in the literature regarding the stigma associated with mental illness, many authors have denounced the lack of studies investigating the phenomenon of stigmatization among psychiatric nurses [17],[29],[30], This phenomenon appears not to be negligible for the weight that mental health nurses (MHNs) have in the recovery process and patient care and for the close relationship with the latter.

### 1.1. Objective of the study

The primary objective of this study is to analyze the degree of stigmatization of mental illness and aptitude for recovery among mental health nurses of the Italian Departments of Mental Health and verify if there is a relationship between these two conditions. The secondary objective is to understand which categories of MHNs are most at risk of having stigmatizing attitudes.

## 2. Materials and methods

### 2.1. Study design and participants

A cross-sectional study was conducted, using an anonymous online questionnaire, among MHNs working in the Italian mental health departments. The period of administration of the questionnaire was between 2022, February 7 and 2022, April 15, all nurses provided their informed consent before participating in the study.

The MHNs were recruited by publishing the proposal for participation in the Diary (n. 46 of February 2022) of the Società Italiana di Scienze Infermieristiche in Salute Mentale (S.I.S.I.M.) and as a post on the Facebook Group of the same society. The Facebook post containing the participation link to the questionnaire, was published on 2022, February 6. The lead author provided his email contact for any doubts or questions from the participants, none of whom felt it necessary to contact him.

This convenient sampling method was chosen to maximize the size of the final sample and the availability/freedom of MHNs to participate in the study. Only and exclusively nurses (including head nurse/managers) who declared to work in Italian mental health services participated in the survey. The questionnaire was not aimed at: nursing students, retired nurses, or nurses who, at the time of completing the questionnaire, were not working in the Italian mental health services.

The responses of 204 mental health nurses were included in the final dataset.

### 2.2. Ethical implications

Before completing the questionnaire, all participants gave their informed consent after an explanation of the purpose of the study. The data was analyzed in aggregate form to guarantee the maximum privacy of the participants. In accordance with Italian legislation, due to the non-interventional nature of this study, authorization from the Ethics Committee was not necessary.

This study was conducted in accordance with the criteria contained in the Declaration of Helsinki [31].

### 2.3. Instrument

The questionnaire consisted of three sections: demographic and occupational information, the Recovery Attitudes Questionnaire-7 (RAQ-7) [32] and the 15-item Open Minds Scale for Health Care Providers (OMS-HC-15) [33].

Socio-demographic and occupational information included: gender, age (expressed in years), marital status, study level, the mental health context in which the nurse is serving and experience (expressed in years) in mental health settings as a MHNs. MHNs were also asked if during their careers they had ever suffered physical aggression from patients or if they had experienced personal or family mental illness. The Recovery Attitudes Questionnaire-7 (RAQ-7) [32] is a self-assessment tool used to determine the respondent's attitudes towards the recovery process from psychiatric disorders. The original version of this scale consisted of 21 items; subsequently transformed into 7 and validated. The 7 items included 2 factors, they are: “Recovery is possible and needs faith” and “Recovery is difficult and differs between people”. Responses to this questionnaire are rated on a 5-point Likert scale (1 = “strongly disagree”, 2 = “disagree”, 3 = “neutral”, 4 = “agree” and 5 = “strongly agree” ), with higher scores indicating a more positive attitude towards the conception of recovery. The RAQ-7, has an acceptable internal consistency, as indicated by Cronbach's α coefficient of 0.70; the overall score of the questionnaire ranges from 5 to 35 points.

The 15-item Open Minds Scale for Health Care Providers (OMS-HC-15) [33], measures the attitudes of health professionals towards people with mental illness. The OMS-HC-15 includes three sub-scales: the first concerns the attitude of health professionals towards people with mental illness (Factor 1), the second concerns the dissemination / search for help in conditions of mental illness (Factor 2) and the third concerns social distance (Factor 3). The internal consistency of OMS-HC-15 items (α = 0.79) and its three subscales (α = 0.67–0.68) were found to be acceptable. The responses to each item on this scale are evaluated on a Likert scale ranging from 1 to 5 points; the overall OMS-HC score can range from 15 (least stigmatizing attitude) to 75 (most stigmatizing attitude) points.

### 2.4. Statistical analysis

SPSS version 20.0 software (IBM Corp., Armonk, New York, United States) was used for data processing.

Descriptive analysis was used to describe the variables considered, frequencies and percentages were used for the counting data. Subsequently, independent *t*-tests (for independent variables with two alternatives) and One-way Anova (for independent variables with 3 or more alternatives) were performed to evaluate the presence of possible differences in the mean of the RAQ-7 and OMS-HC-15 scores in relation to the socio-demographic and professional variables considered. Finally, two stepwise linear regression models were applied to evaluate whether the socio-demographic and professional variables considered were predictive of different attitudes both on the concept of recovery and in the stigmatization of mental pathology. The results of stepwise linear regression models were reported as Beta coefficients and related 95% confidence intervals.

## 3. Results

A total of 204 nurses from the Italian Departments of MHNs participated in the study, Table 1 shows the demographic characteristics of the sample; most participants were female (65.2%) over the age of 50 years old (42.2%) and married (46.1%). Among the participants, 44.6% worked at a General Hospital Psychiatric Unit (GHPU) and 35.3% of them had experience in mental health services over 20 years; from our analysis, it emerges that most of the MHNs had basic training (Regional School Diploma / Bachelor of Science in Nursing) and that 64.2% of participants had experienced at least one episode of physical aggression by a patient in their career. 61.8% of MHNs said they had never had a personal or family experience of mental illness.

### 3.1. Recovery attitude

Administration of the Recovery Attitudes Questionnaire-7 (RAQ-7) showed an overall mean score among the population of MHNs studied of 28.93 points (*SD* = 4.18).

Table 2 shows that the average score obtained by MNHs on the RAQ-7 scale shows statistically significant differences (*p* < 0.001) in relation to the qualification held by these operators. In particular, MHNs with advanced training (First Level Master, MNS or PhD) were matched with average scores on the questionnaire, which were higher expressions of a greater capacity for recovery. In addition, nurses working in the Departments of Mental Health (DMHD) also reported extremely high scores compared to colleagues working more closely with patients.

Linear regression (Table 3 and Table 4), showed how the average score obtained on the OMS-HC scale, which expresses the attitude of respondents towards patients with mental illness (and the degree of stigmatization of the same), was the only one predictor, among those proposed in this study, able to significantly modify (*p* < 0.001) the aptitude for recovery.

**Table 1.** Sample characteristics (204 MHNs).

Program	Category	*N*	Proportion
Gender	Female	133	65.2%
	Male	71	34.8%
Age (year)	<30	24	11.8%
	30–40	39	19.1%
	40–50	55	27.0%
	>50	86	42.2%
Marital status	Unmarried	77	37.7%
	Married	94	46.1%
	Divorced	31	15.2%
	Widower	2	1.0%
Care setting	Community Mental Health Centre (CMHC)	53	26.0%
	Direction of Mental Health Department (DMHD)	9	4.4%
	General Hospital Psychiatric Unit (GHPU)	91	44.6%
	Residential Facilities (RF)	51	25.0%
Study level	Regional School Diploma	51	25.0%
	BnS	62	30.4%
	MnS/PhD	26	12.7%
	First/Second level University Master	65	31.9%
Experience in mental health setting (year)	0–5	56	27.5%
	6–10	35	17.2%
	11–20	41	20.1%
	>20	72	35.3%
Have you ever been physically assaulted by a patient?	No	73	35.8%
	Yes	131	64.2%
Have you ever had personal / family experience of mental illness?	No	126	61.8%
	Yes	78	38.2%

### 3.2. Stigmatization of mental illness

Administration of the Open Minds Scale for Health Care Providers at 15 items (OMS-HC-15), showed an overall mean score among the population of MHNs studied of 33.21 points (*SD* = 8.83).

From the comparisons conducted on the averages (see Table 4), we have noticed how some variables could be decisive in modifying the attitude towards mental illness, in particular: marital status, working in one setting rather than another, the level of studies and years of experience were significant in the *F*-test. We observed that mental health nurses: widowers, workers at Community Mental Health Centres (CMHC), with advanced study level (University Master/MnS/PhD) and with more years of experience showed less levels of stigmatization towards users with the disease mental, compared to their respective colleagues.

**Table 2.** RAQ 7 items-factors and total scores by group characteristics (*Mean* = 28.93, *SD* = 4.18).

Variable		Mean (*SD*) Factor 1_RAQ-7	Mean (*SD*) Factor 2_RAQ-7	Mean (*SD*) RAQ-7	*t*/*F* (7-items)	*p* value (7 items)
Gender	F	16 (2.5)	12.8 (1.7)	28.8 (3.7)	-0.49	0.61
	M	16.2 (2.9)	12.8 (2.2)	29.1 (4.9)		
Age (y)	<30	15.3 (3.2)	12.5 (2.8)	27.8 (5.8)	1.09	0.35
	30–40	15.7(2.4)	12.5 (1.7)	28.3 (3.7)		
	40–50	16.4(2.4)	12.7 (1.7)	29.1 (3.8)		
	>50	16.2 (2.7)	13 (1.7)	29.3 (4)		
Marital Status	Unmarried	16.1 (2.6)	12.7 (2.1)	28.9 (4.3)	0.23	0.87
	Married	15.9 (2.6)	12.7 (1.7)	28.7 (4)		
	Divorced	16.4 (2.9)	13 (1.7)	29.4 (4.3)		
	Widower	16 (4.2)	13.5 (2.1)	29.5 (6.3)		
Setting	CMHC	16 (2.7)	12.8 (1.7)	28.8 (4)	0.84	0.47
	DMHD	17.1 (2.9)	13.4 (2.4)	30.5 (5.2)		
	GHPU	15.8 (2.9)	12.7 (2)	28.5 (4.6)		
	RF	16.5 (1.9)	12.8 (1.5)	29.3 (3.1)		
Study Level	Regional School Diploma	16.1 (3)	12.6 (1.7)	28.7 (4.3)	6.38	<0.001
	BnS	15 (2.7)	12.1 (2.2)	27.2 (4.5)		
	MnS/PhD	16.5 (2.6)	13.5 (1.4)	30 (3.8)		
	First/Second level University Master	16.8 (2)	13.3 (1.5)	30.1 (3.1)		
Experience in Mental Health Setting (y)	0–5	15.4 (2.5)	12.4 (2.1)	27.9 (4.4)	2.01	0.11
	6–10	16.5 (2.6)	13 (1.6)	29.5 (3.9)		
	11–20	15.8 (2.9)	12.7 (1.7)	28.6 (4.2)		
	>20	16.5 (2.6)	13 (1.8)	29.5 (4)		
Have you ever been physically assaulted by a patient?	No	16.3 (2.4)	13.1 (1.5)	29.5 (3.6)	2.30	0.13
	Yes	15.9 (2.8)	12.6 (2)	28.6 (4.4)		
Have you ever had personal/family experience of mental illness?	No	15.9 (2.7)	12.9 (1.9)	28.8 (4.2)	0.70	0.79
	Yes	16.3 (2.5)	12.6 (1.8)	29 (4)		

Note: In this study, the Cronbach alpha coefficient (7-items) was found as 0.761.

**Table 3.** RAQ-7 items-linear regression analysis.

Predictor	*B*	*t*	*Sign*.	95% *CI*	
				Lower	Higher
(Costant)	36.104	35.370	<0.001	34.091	38.116
OMS-HC-15 items Score	-0.216	-7.274	<0.001	-0.275	-0.158

Note: Dependent variable: Total Score_RAQ-7. Model summary: *R* = 0.456, *R^2^* = 0.208, adjusted *R^2^* = 0.204

The linear regression, conducted on all the socio-demographic and professional variables selected for this study (Table 5 and Table 6), showed in its most performing model (Model 4), as the score obtained in the RAQ-7, the marital status, the setting in which one works, and the level of education may be significant predictors of the degree of stigmatization of mental illness among the mental health nurses studied. In general, having suffered physical assaults in psychiatric settings or having had personal or family experience of mental illness did not appear suggestive of more stigmatizing attitudes or a lower aptitude for recovery.

### 3.3. Relationship between stigmatization level and recovery attitude

Our correlation analysis between the scores on the OMS-HC-15 scales (attitude and stigma towards mental illness) and RAQ-7 (aptitude for recovery) showed a Pearson correlation index (ρ) = -0.456 (*p* < 0.001).

**Figure 1.**
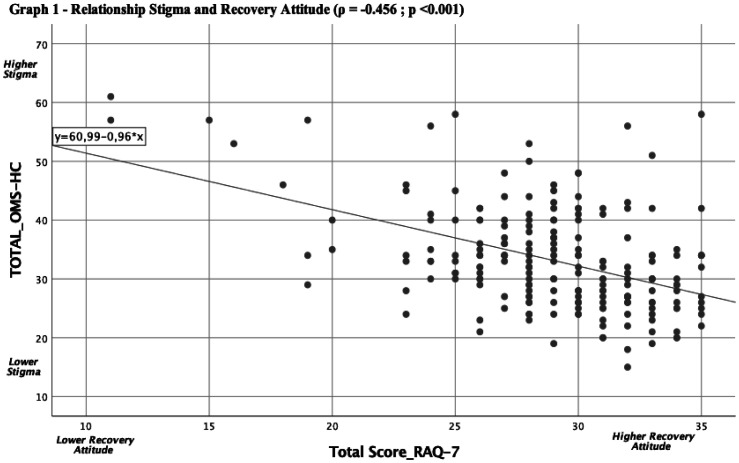
Relationship stigma and recovery attitude.

In general, the mean scores obtained by MHNs in the two scales were inversely proportional (see Figure 1); it should be remembered that the two scales have an opposite connotation, i.e., in RAQ-7 as the scores increase there is a more positive attitude towards recovery (and vice versa) and the OMS-HC-15 when scores decrease a less stigmatizing view of mental illness (and vice versa).

## 4. Discussion

Stigmatization and discrimination represent a great barrier to the healing process of the individual with mental illness, in this study we tried to explore the attitude to recovery and the tendency to stigmatization towards users with mental illness among the nurses of the Italian Departments of mental health, using the RAQ-7 and OMS-HC-15 scales.

It was observed that the average scores obtained by MHNs (*N* = 204) in the two aforementioned scales, showed an inversely proportional trend; it should be remembered that the two scales have an opposite connotation, i.e., in RAQ-7 as the scores increase there is a more positive attitude towards recovery (and vice versa) and the OMS-HC-15 when scores decrease a less stigmatizing view of mental illness (and vice versa).

From the results coming from the administration of the RAQ-7, it emerged that the aptitude for recovery can be influenced by the level of study of the nurses, this being able, when it goes beyond basic training, to determine more positive attitudes. Similarly, previous studies had observed how advanced training is a factor capable of triggering and promoting positive attitudes towards the recovery process in mental health [34]. Moreover, past studies, had hypothesized how even the characteristics of basic training can play a decisive role in relation to stigma, for example a study conducted in Poland, had observed how stigmatizing attitudes emerge in the phase of education, or in any case before to start a professional career [35].

**Table 4.** OMS-HC 15 items-Subscales and total scores by group characteristics (*Mean* = 33.21; *SD* = 8.83).

Variable		Mean (*SD*) Attitude	Mean (*SD*) Social Distance	Mean (*SD*) Disclosure	Mean (*SD*) 15-items	*t*/*F* (15-items)	*p* value (15-items)
Gender	F	10.8 (4.4)	10.1 (2.8)	11.3 (3.5)	32.3 (8.2)	-1.84	0.06
	M	12.8 (5)	10.7 (3.2)	11.1 (3.6)	34.7 (9.7)		
Age (y)	<30	11.5 (5.3)	11 (3.3)	13 (3.4)	35.6 (10.1)	1.4	0.24
	30–40	11.9 (4.7)	11.2 (3)	11.5 (3.7)	34.6 (9.2)		
	40–50	11.4 (4.3)	10.1 (2.8)	10.6 (3.4)	32.2 (8)		
	>50	11.3 (4.9)	10.0 (2,8)	11(3.4)	32.4 (8.6)		
Marital Status	Unmarried	12.3 (5.6)	11.4 (2.9)	11.7 (3.7)	35.4 (9.8)	2.99	0.03
	Married	11.3 (4)	9.7 (2.7)	11.1 (3.1)	32.2 (7.5)		
	Divorced	10.3 (4.2)	9.9 (2.9)	10.7 (4)	31 (9)		
	Widower	11.5 (0.7)	5 (0)	11.5 (3.5)	28 (4,2)		
Setting	CMHC	10.3 (3.5)	9.7 (2.9)	10.4 (3)	30.4 (7.1)	3.3	0.02
	DMHD	10.3 (2.5)	10 (1.9)	10.4 (3)	30,8 (5.7)		
	GHPU	12.4 (5.4)	10.7 (3.2)	11.8 (3.7)	34.9 (9.9)		
	RF	11.5 (4.7)	10.6 (2.8)	11.5 (3.7)	33.6 (8.2)		
Study Level	Regional School Diploma	12.5 (5.5)	10.2 (3.1)	11.1 (3.1)	33.8 (8.9)	4.56	<0.01
	BnS	12.2 (4.9)	11.1 (3.1)	12.6 (39)	35.9 (9.6)		
	MnS/PhD	11.7 (3.3)	10.4 (2.7)	108 (3.6)	32.8 (7.2)		
	First/Second level University Master	10.1 (4.2)	9.8 (2.8)	10.4 (3.2)	30.3 (7.8)		
Experience in Mental Health Setting (y)	0–5	12.1 (4.8)	11.3 (2.9)	12.8 (4)	36.1 (9.5)	2.96	0.03
	6–10	10.9 (4.8)	10.5 (2.8)	10.4 (2.8)	31.9 (7.8)		
	11–20	11.5 (4.1)	10 (3.1)	11.2 (3.4)	32.8 (8.1)		
	>20	11.4 (5.1)	9.9 (3)	10.6 (3.3)	31.8 (8.8)		
Have you ever been physically assaulted by a patient?	No	10.9 (4.7)	10 (2.7)	10.9 (3.4)	31.9 (8.1)	-1.51	0.132
	Yes	11.8 (4.7)	10.5 (3.1)	11.4 (3.6)	33.9 (9.1)		
Have you ever had personal/family experience of mental illness?	No	11.6 (5.3)	10.3 (3.1)	11.1 (3.8)	33.3 (10)	0.35	0.72
	Yes	11.3 (3.6)	10.4 (2.7)	11.3 (3)	32.9 (6.4)		

Note: s study, the Cronbach alpha coefficient (15-items) was found as 0.797.

In addition, according to some authors [36], the clinical practice phase, thanks to the proximity and care of people with mental health problems, would improve attitudes and attitudes towards mental health in nursing students who have not had mental health problems and also in younger students.

**Table 5.** OMS-HC 15 items-linear regression analysis.

Model	Predictor	*B*	*t*	*Sign*.	B 95% *CI*	
					Lower	Higher
1	(Constant)	60.987	15.805	<0.001	53.379	68.596
	RAQ-7 items-Score	-0.960	-7.274	<0.001	-1.221	-0.7
2	(Constant)	64.692	16.270	<0.001	56.852	72.532
	RAQ-7 items-Score	-0.948	-7.324	<0.001	-1.204	-0.693
	Marital status	-2.258	-3.027	<0.01	-3.729	-0.787
3	(Constant)	61.069	14.744	<0.001	52.902	69.237
	RAQ-7 items-Score	-0.953	-7.471	<0.001	-1.205	-0.701
	Marital status	-2.099	-2.848	<0.01	-3.552	-0.645
	Setting	1.291	2.685	<0.01	0.343	2.240
4	(Constant)	61.626	14.962	<0.001	53.504	69.748
	RAQ-7 items-Score	-0.9	-6.969	<0.001	-1.155	-0.646
	Marital status	-2.083	-2.848	<0.01	-3.525	-0.641
	Setting	1.383	2.885	<0.01	0.438	2.328
	Study Level	-0.937	-2.036	0.04	-1.844	-0.03

Note: Dependent variable: Total Score OMS-HC.

**Table 6.** Model summary of table 5.

Model	*R*	*R^2^*	Adjusted *R^2^*
1	0.456	0.208	0.204
2	0.492	0.242	0.235
3	0.518	0.269	0.258
4	0.532	0.283	0.269

Conversely, it should be emphasized that in the last two decades, psychiatric nursing contents have been gradually eroded in educational programming [37] with some reservations regarding the timing of learning [38].

The ANOVA analysis conducted in our study showed how the level of studies can also significantly influence the global score of the OMS-HC-15 scale. However, the variables: marital status, setting and longevity of service in mental health departments could provide insights for future research. Generally, it was noted that widowed nurses working at CMHCs with an advanced level of training and with more years of experience showed lower levels of stigmatization of mental illness than their colleagues Although some authors [11],[39] have previously highlighted the tendency of the older general population to have more stigmatizing attitudes towards people with mental illness, this study did not find such evidence on the nursing sample studied, but rather did it is observed that nurses with more years of experience in mental health may instead show less stigmatizing attitudes than colleagues with less experience. This appears in contrast to that present in a transversal study conducted in Singapore [40], where health workers (both doctors and nurses) with experience of more than 10 years of service in psychiatry, would be more stigmatizing towards psychiatric patients, due to prolonged exposure to associative stigma by their colleagues.

With respect to marital status, it is plausible that the reduced presence of widower respondents may have led to a distortion of global scores (bias), just as it can be assumed that a possible di-psychological stress experienced by these operators following the death of their loved one (complicated bereavement), may have produced in these a direct contact with mental illness and consequently have reduced their levels of stigma.

In our research, having had personal or family experience of mental illness did not appear suggestive of more stigmatizing attitudes or a lower aptitude for recovery, previously, according to a qualitative research conducted in Indonesia [41], the relatives of patients with psychiatric pathology would still tend to maintain a stigmatizing attitude towards mental illness and patients suffering from it. Although it has emerged that the Eastern realities are more stigmatizing than the Western ones, in Indonesia relatives can change their domicile for shame or for not being traced. It is likely that the documented impacts on families of people with mental illnesses, including sleep disturbances, changes in interpersonal relationships, worsening well-being and reduced quality of life, play a role in the approach of family members to people with mental illness.

In addition, it might be interesting to investigate the differences that emerged in the scores on the OMS-HC-15 scale, between nurses who provide their assistance at GHPUs compared to colleagues engaged in territorial assistance (CMHC). In this sense, it could be assumed that the continuous proximity to acute psychiatric situations and the lack of direct contact with the patient's daily reality could stimulate more stigmatizing attitudes among the MHNs who assist patients in this phase of the disease. On the contrary, being able to appreciate the person's moments of well-being with a certain continuity, in fact, could lead to a set of positive attitudes that could explain the differences observed in this study.

However, the issue of stigma is certainly influenced by cultural beliefs, and not only because it has been shown that members of e.g., Eastern cultures may have a higher degree of stigma than Westerners [42], but also because of significant differences between individual countries can arise from many aspects, including gender distribution and age [43]. For example, a study conducted in China showed an improvement in the attitude among medical students towards patients with mental illness after the training period [44].

Finally, linear regression, applied to the overall score of the OMS-HC-15 scale (*Table 5*), showed in its most performing model, how the score obtained in the RAQ-7, the marital status, the setting in which one works and the level of education may be significant predictors of the degree of mental illness stigmatization among the mental health nurses studied.

In the past, other authors have highlighted how negative attitudes, such as stigma and poor recovery aptitude, can have consequences on health care outcomes and more [45], the implications can be both subjective and objective.

Some patients, in fact, reported feeling ignored, judged inadequate to tolerate their physical symptoms [46] with the consequent risk of developing self-stigma. This makes the person affected by a mental illness feel inferior, abnormal, leading to attitudes of renunciation; therefore, to isolate himself even more from society [47]. The patient's search for help is suppressed, thereby aggravating the severity of the disease [48].

In fact, assistance characterized by stigmatizing attitudes and a poor aptitude for recovery results in the repeated under-treatment of physical symptoms [49], in a barrier for building a relationship with these patients and a reduced pharmacological and therapeutic compliance. In general [50].

### 4.1. Limitations

The responses provided by the participants may have been subject to biases of social desirability.

Within the limits of this work there is a lack of evaluation of sample power, in fact, although the sample of the study was large and diverse, for the selection method chosen by the researchers to recruit participants (sampling of convenience also through the use of Facebook) the representativeness of the sample could be doubtful and not generalizable. Another bias related to the selection process is the exclusion from participation in the study of all those mental health nurses not enrolled in Società Italiana di Scienze Infermieristiche in Salute Mentale (S.I.S.I.M.) or Facebook.

## 5. Conclusions

This study, conducted on Italian mental health nurses, showed how the latter's attitude towards recovery is closely related to the tendency to stigma towards people with mental illness; being in possession of advanced training seems to be a factor capable of affecting both the tendency of MHNs to stigma of mental illness and the aptitude for recovery, resulting in better care outcomes in the field of mental health nursing. The setting in which one serves (hospital / territorial) seems to be also a determining variable in influencing the tendency to stigma, as well as the longevity of service in mental health.

This study could assist nursing managers, leaders or educators in making decisions about managing and preventing stigma among MHNs; although the average level of stigma and attitudes towards recovery were generally acceptable, there is room for improvement, especially in basic training. In fact, a re-orientation of university curricula and a propensity, always in this area, oriented above all to direct contact rather than theoretical education might be desirable. In addition, it would be interesting to understand whether a rotation of the nursing staff between intensive (hospital) and community care could lead to a reduction in stigma among these professionals and ensure a more complete vision of the patient care process. Mental health nurses, along with the mass media, can be powerful testimonials of de- stigmatization. Finally, it is likely that anti-stigma campaigns at national level should be strengthened in Italy.
